# Damiana—Unexpected Results from Its Use in a Case

**Published:** 1882-11

**Authors:** W. H. Bently


### DAMIANA—UNEXPECTED RESULTS FROM ITS USE IN A CASE.

I am by no means a believer in the marvelous, and I have an aversion to the sensational, and especially in medicine. The following case seems to me so remarkable, however, that I venture to lay it before the readers of the GAZETTE :

Mr. A. is about 65 years of age. He has been a man of great physical powers and endurance. From boyhood until some four years since he has been a very diligent and hard laborer. Several years he spent in rafting timber and flat-boating. During these years much of his time was spent in camp or under the shelving rocks along the uninhabited banks of a mountain stream, where he constructed his timber rafts. He says that he never stopped working on account of rain or snow, and he kept up this practice on his farm, doing his milking and going most of his errands during night time.

In January, 1878, he had an attack of pneumonia, his first illness. It was a severe and protracted case, and three or four months his physicians and friends were in constant despair of his life. After a time he got able to go about his farm, but just then general dropsy supervened. The case was quite intractable, and after a few months of almost fruitless treatment, I was requested to take charge of it. It yielded very tardily to my treatment, but by November had entirely disappeared. He had not regained his health, however. His skin was sallow and waxy ; his tongue coated at the base with a dirty whitish fur ; bowels torpid ; digestion feeble ; appetite capricious. He was under treatment for this condition most of the ensuing winter. By spring he was able to ride about the country and to superintend his extensive and diversified business.

In May, 1879, he got on a "big spree" in town, which lasted a week, riding home—twelve miles—at the end of the time, all the way through a drowning rain. He now had a return of his dropsy, which seemed more intractable than before. This was followed by the same train of morbid symptoms that was the sequel to his first case.

The year 1880, with the exception of the " big drunk," is a repetition of the history of his case of the previous year.

The early spring of 1881 found his old enemy in prompt attendance and more obstinate than ever. I concluded to resort to *paracentesis*, and had appointed a day for the operation. The night previous to the day fixed he had an alarming attack of cholera morbus. This yielded reasonably well under treatment. The dropsy had wholly disappeared, and for a time it seemed that he would soon regain his health.

Two months later (about Sept. 1st), chronic diarrhœa set in, which, for more than three months, baffled my every effort. During the time I had recourse to quite a number and variety of drugs. As his nervous system seemed much prostrated, I gave him some comp. phosphorus pills. Under their use he improved for a time ; then they disagreed with his stomach, and were discontinued.

One day while visiting him, I prescribed for him four ounces of the fluid extract of damiana to be taken in the usual doses. I had observed in several cases in which I had exhibited it for other purposes, it had greatly benefited the enfeebled digestion. I thought this was produced through the nervous system, and I reasoned that if it could impart vigor to the generative organs, it must act through the spinal column. I had noticed, too, that the use of damiana nearly always invigatored the bladder, the kidneys, and, in fact, the entire urinary apparatus. Hence, although I had small hope in this case, I did not prescribe it as a mere placebo. The patient began to improve almost immediately. After taking the four ounces, I prescribed a pound. He was to all appearances well before he had used all this, but I persuaded him to continue its use until an additional pound had been taken.

He is now as vigorous as any man of his age could reasonably expect to be. He informed me that he had been entirely impotent for ten or twelve years, but he declares that he has fully recovered in this respect, and adds that it is difficult for him to restrain himself within what he considers reasonable bounds.

It is probable that a trial of the drug in a hundred similar cases would not achieve, in a single instance, so complete a triumph. Yet, I think it worthy of trial. I think, too, that this case throws some light on the *modus operandi* of this highly valuable agent.

The preparation used throughout this case was that of Parke, Davis & Co.

The dose was a fluid drachm before meals and at bed-time.

Aug. 1st, 1882.—W. H. Bently, in *Therapeutic Gazette*.

